# Agrobacterium-mediated co-transformation of American Chestnut (*Castanea dentata*) somatic embryos with a wheat oxalate oxidase gene

**DOI:** 10.1186/1753-6561-5-S7-O43

**Published:** 2011-09-13

**Authors:** Bo Zhang, Andrew Newhouse, Linda McGuigan, Charles Maynard, William Powell

**Affiliations:** 1 State University of New York-College of Environmental Science and Forestry (SUNY-ESF), USA

## Background

American chestnut is a tree of great historical, ecological, and economical importance. It once dominated forests in eastern United States until the introduction of chestnut blight fungus (*Cryphonectria parasitica*) in the late 19th century. Within 50 years, *C. parasitica* killed almost all of the 4 billion American chestnut trees in the eastern United States. The fungus first infects wounded stem, secretes oxalic acid to decrease the pH of the infected tissue to toxic levels for the tree, but optimum for fungal enzymes, and then mycelia fans spread forming a canker which when it girdles a branch prevents water and nutrient transport, eventually killing the tree above the canker. The fungus does not infect the roots, thus allowing the growth of adventitious shoots to keep the tree alive. However, this survival is only temporary because these spouts will again get infected by the fungus and die back to the ground. It is this continuing circle that made American chestnut, once a great canopy tree, to no more than an early-succession-stage shrub today. Efforts to restore American chestnut back to its native range are currently being made. In our labs, we use an *Agrobacterium*-mediated co-transformation system to deliver potential resistant genes into somatic embryos of American chestnut together with a selectable marker gene. The gene of interest we used is an oxalate oxidase gene from wheat (*Triticum aestivum*). Oxalate oxidase (OxO) can degrade the fungus secreted oxalic acid to carbon dioxide and hydrogen peroxide. This action has a duel-effect: bringing up the pH of the infected sites thus neutralizing the virulent effect of oxalic acid, and increasing the expression of defense related genes induced by hydrogen peroxide byproduct. The selectable marker we used is Green Florescent Protein (GFP) from *Aequorea victoria*,which allows us to have a direct visual selection of successful transformants.

## Methods

**Figure 1:**
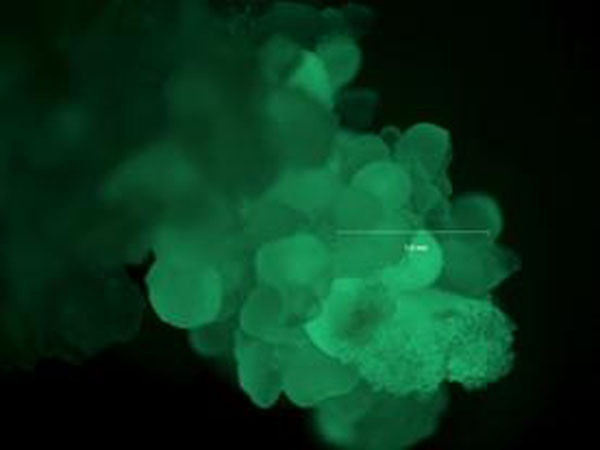
GFP expression in transformed American chestnut embryo cluster under UV light.

*Agrobacterium tumefaciens* strain EHA105 was used for the co-transformation. Vectors containing OxO and GFP were introduced to EHA105 separately via electroporation so each *Agrobacterium* had only one vector. Transformed EHA105 were grown separately under the selection of kanamycin and rifampicin until an O.D. of 0.8-1.2 was reached. *Agrobacterium* with optimum O.D. were harvested and re-suspended in *vir* induction medium containing acetosyringone. To approximately equalize the concentration of bacteria samples with different vectors, the amount of *vir* induction medium added was calculated by multiplying the O.D. value with 50ml. Bacteria samples were incubated in *vir* induction medium for three to four hours under room temperature with slow agitation. After *vir* induction, bacteria samples containing different vectors were mixed together in two different volume ratios: 2 (OxO):1 (GFP) and 4 (OxO):1 (GFP). The mixture was then added to embryo clusters to bring a total volume of 6ml followed by co-cultivation on a turning machine for an hour at room temperature. After the co-cultivation, embryo clusters were taken out to put in desiccation plates (plates with 200ul ddH_2_O saturated filter paper) and kept in dark for two days under room temperature. After desiccation, embryos were transferred to *Agro*-kill medium containing carbenicillin and cefotaxime and kept in dark under room temperature. After a week, embryos were transferred to selection medium containing carbenicillin, cefotaxime, and appropriate selection agents (herbicide PPT and paromomycin in our case). Embryos were transferred to fresh selection medium every two weeks and were examined for GFP expression under a UV microscope (Figure 1).

GFP positive embryos were separated and multiplied in selection medium followed by genomic DNA extraction and PCR. Positive transformants with both GFP and OxO together with six GFP-only events were selected for regeneration as previously developed in our labs. The expression and copy number of OxO were tested by RT-PCR (Real Time-PCR).

## Results and discussion

Thirteen experiments with a total of 208 embryo clusters were done using the 2 (OxO):1 (GFP) ratio resulting in 50 GFP positive events. 10 out of the 50 events were also OxO positive, giving a co-transformation efficiency of 20%, or 4.8% per embryo cluster. Three experiments with a total of 150 embryo clusters were done using the 4 (OxO): 1 (GFP) ratio resulting in 45 GFP positive events. 18 out of the 45 events were also OxO positive, giving a co-transformation efficiency of 40%, or 12% per embryo cluster. Doubling the ratio of vector containing gene of interest to vector containing the selectable marker from 2:1 to 4:1 gave a 2.5-fold increase in co-transformation efficiency. All of the 28 events went through the regeneration process together with 6 GFP-only events. 4 out of the 10 events produced by 2:1 ratio regenerated into whole plants. 17 out of the 18 events produced by 4:1 ratio regenerated into whole plants. All 6 GFP-only events regenerated into whole plants. These regenerated American chestnut plants seem to exhibit normal morphology. A gradient of OxO expression levels were found with the highest being more than 200 fold higher than the lowest expressed event. Copy numbers of the transgene *oxo* were also tested via RT-PCR showing most events with 2 copies, few with single copy, and a couple with higher copy numbers. The difference in regeneration ability and expression levels among these events was likely a result of position effect, which the expression of foreign gene is affected by the insertion site and nearby regulatory factors in the genome.

## Conclusions

The efficiency of *Agrobacterium*-mediated co-transformation of American chestnut somatic embryos improved significantly when increasing the ratio of vector containing the gene of interest to vector containing the selectable marker in the co-cultivation mixture. Other factors of co-transformation including *Agrobacterium* strain, co-cultivation time, and desiccation time, etc. can also be investigated to improve the overall efficiency of this process. Co-transformation proved to be an effective way to introduce foreign genes into American chestnut to test their effectiveness on chestnut blight resistance. Having GFP on a separate plasmid provided a useful tool for removing of the marker gene in future. However, further tests need to be done to determine whether the GFP gene is linked to *oxo* in the genome.

